# Multivariate analysis of newly diagnosed hip, knee, and combined hip and knee Osteoarthritis and recurrent fall risk: data from the Osteoarthritis Initiative

**DOI:** 10.3389/fmed.2026.1710951

**Published:** 2026-04-10

**Authors:** Reza Sorbi, Ghaith Al Tawil, Simone Gantz, Baraa Khamees, Maciej Simon, Babak Moradi, Hadrian Platzer

**Affiliations:** 1 Department of Orthopedics and Trauma Surgery, University Medical Center Schleswig-Holstein, Kiel, Germany; 2 Orthopedic Research Center, Kiel University, Kiel, Germany

**Keywords:** aging, depression, falls, osteoarthritis, physical function, risk factor

## Abstract

**Background:**

Osteoarthritis (OA), the most prevalent joint disease, is associated with impaired mobility and may contribute to fall risk in older adults. Recurrent falls (≥two falls/year) are of particular concern due to their impact on morbidity and independence. This study is the first to examine whether individuals with early hip and/or knee OA are at increased risk of recurrent falls within 12 months of diagnosis and to identify biopsychosocial factors associated with fall risk.

**Methods:**

Data were derived from the Osteoarthritis Initiative (OAI), a retrospective cohort of 4,427 participants stratified into four groups: individuals without OA, with knee OA, with hip OA, and with combined hip and knee OA. Self-reported recurrent falls within 12 months post-diagnosis were analyzed. Correlation and multivariable logistic regression analyses were conducted to identify predictive factors and interactions.

**Results:**

The presence of OA alone was not independently associated with recurrent falls in short term. However, multivariable logistic regression identified several factors associated with recurrent falls. There was a trend toward increased odds among participants with hip OA (*OR* = 2.35, *p* = 0.062). Individuals under 65 years had lower odds of recurrent falls compared to older adults (*OR* = 0.752, *p* = 0.034), and better physical function was protective (SF-12: *OR* = 0.980, *p* = 0.005). Depressive symptoms were associated with increased odds (CES-D: *OR* = 1.024 per point, *p* = 0.005). Interaction analysis showed that depression particularly increased recurrent falls risk in those with knee OA (*OR* = 1.036, *p* = 0.034), while younger age was protective among individuals with hip OA (*OR* = 0.230, *p* = 0.036).

**Conclusions:**

While Osteoarthritis itself does not directly predict recurrent falls in short term, its risk in individuals with OA is shaped by a multifactorial interplay of age, marital status, ethnicity, physical functioning, and depressive symptoms, with specific interactions involving OA location. Therefore, a differentiated assessment and multidisciplinary approach addressing these factors are essential to reduce recurrent falls in this population.

## 1 Introduction

Osteoarthritis is the most prevalent degenerative joint disease worldwide, primarily affecting weight–bearing joints such as hips and knees (1), and is characterized by chronic pain, stiffness, muscle weakness (2, 3). These impairments frequently lead to reduced mobility, impaired proprioception, and diminished balance control (4–6), all of which are established contributors to fall risk.

Falls represent a major public health concern in aging societies. Approximately one-third of adults aged 65 years and older experience at least one fall annually (7), and 15% experiencing recurrent falls (8) (≥two falls/year), which are associated with functional decline, hospitalization, and loss of independence. Some studies have linked both radiographic severity and symptomatic OA with elevated fall risk (9, 10), while others have reported increased fall risk linked to symptomatic OA (11, 12). However, OA-related fall risk is not solely determined by joint pathology; it is further compounded by comorbidities (13–16) and age-related health conditions (17–19), which may exacerbate functional decline, compromise balance and muscle strength, and further increase fall susceptibility (4, 20). Each additional chronic condition is estimated to increase fall rates by approximately 7%, and individuals with three or more chronic conditions experience a 21% higher incidence of falls over time (21). Moreover, clinical and psychological factors intrinsic to OA play a significant role in fall risk. A cross-sectional study of 372 individuals with knee OA found that those with greater symptom severity, as measured by Knee Injury and Osteoarthritis Outcome Score (KOOS) pain and disability scores, had significantly higher odds of having experienced a fall (22). Fear of falling is also highly prevalent in OA populations and has been shown to independently predict future falls. For instance, over 80% of women with knee OA in one study reported significant fear of falling, which was closely associated with pain and previous falls (23). Psychological conditions such as depression and anxiety, which are frequent in OA, have been linked to impaired motor coordination, reduced physical activity, and increased frailty, factors that further elevate fall risk (19, 24).

Despite increasing recognition of the link between OA and falls, most prior studies have focused on isolated clinical, psychological, or demographic factors, often considered hip or knee OA separately rather than a comprehensive OA perspective. Moreover, recent studies lack an integrative framework that captures the multidimensional nature of fall risk in OA.

To our knowledge, this is the first study to investigate the recurrent fall risk in hip, knee, or combined OA compared to those without OA within a 12-month follow-up, explicitly integrating physical, psychological, and social determinants in a unified biopsychosocial framework. By applying this comprehensive approach, this study seeks to generate a more comprehensive understanding of fall risk in OA and novel insights, which may improve individual risk stratification and inform targeted prevention strategies for recurrent falls in the context of OA and aging.

## 2 Materials and methods

This study is a retrospective analysis of data from the Osteoarthritis Initiative database (https://nda.nih.gov/oai), a large-scale, multicenter, longitudinal cohort study. The OAI commenced participant recruitment in 2004 to conduct a longitudinal investigation into osteoarthritis. The initial data collection was performed in 2006, followed by subsequent assessments at systematically scheduled intervals. The Osteoarthritis Initiative is a longitudinal cohort study that enrolled a total of 4,797 participants across four clinical centers in the United States. The study protocol received ethical approval from the institutional review boards of all participating sites, as well as from the coordinating center at the University of California, San Francisco. Prior to enrollment, written informed consent was obtained from all individuals.

Our inclusion and exclusion criteria strictly follow the methodology outlined on the official website of the OAI (https://nda.nih.gov/oai/study-details). Participants were classified as having OA if they reported a physician-diagnosed symptomatic hip or knee OA within the 12 months preceding data collection, confirmed by radiographic evidence. The individuals without OA comparison group consisted of OAI participants at high risk for developing OA but without a prior diagnosis. The OAI defines ‘high risk' for developing osteoarthritis based on various factors that may increase the risk individually or in combination, including age over 45, presence of knee or hip pain or symptoms, overweight or obesity, prior knee injuries or surgeries, family history of OA or knee replacement, presence of Heberden's nodes, regular activities involving repeated knee bending, and the use of medication to treat OA symptoms. In the Osteoarthritis Initiative, a fall is defined as an event in which a person unintentionally comes to rest on the ground or a lower level. This definition is consistent with the traditional clinical definition of a fall and is used in the OAI to examine the frequency and risk factors associated with falls among participants. All OAI participants were asked whether they had experienced a fall if they answered “yes” to the question regarding falls within the previous 12 months. A recurrent fall was defined as experiencing two or more falls within a 12-month period.

Data extraction was performed between February 2022 and June 2022.

The analyses conducted refer to the baseline dataset as well as the 12–month follow–up data. The short-term observation period was deliberately chosen to reflect a clinically relevant timeframe and to minimize recall bias, which is particularly relevant in older adults. A descriptive analysis was performed to characterize the study population based on a comprehensive set of demographics, clinical, functional, and psychosocial predictors were included in the model.

Of the 4,797 participants originally enrolled in the Osteoarthritis Initiative, 4,427 were included in the present analysis. Participants were excluded primarily due to missing baseline information on key covariates, incomplete 12-month follow-up data on falls, or insufficient data to reliably classify osteoarthritis status. A complete-case approach was applied for all analyses. Accordingly, participants with missing values in the outcome variable, covariates, or interaction terms required for the multivariable models were excluded from the respective analyses. No imputation of missing data was performed.

Continuous variables were summarized as means with standard deviations and included age, BMI, Charlson Comorbidity Index, SF−12 Physical Component Summary score, KOOS Quality of Life subscale, KOOS Function in Sports and Recreational Activities subscale, and the CES-D depression score. The distribution of continuous data was assessed using Shapiro–Wilk test.

Categorical variables such as sex, ethnicity, income, marital status, smoking status, OA knee, OA hip, combined hip and knee OA, and history of falls were presented as absolute numbers and percentages.

To examine associations between categorical variables, Chi-Square tests were applied. Correlational analyses were performed to explore potential relationships between study variables. When variables were not normally distributed or non-linear relationships were assumed, Spearman's rank correlation (ρ) was used. Associations between dichotomous and continuous variables were examined using the point-biserial correlation (r), a special case of the Pearson correlation (see Supplementary Table). These correlation analyses were additionally used as a statistical preselection step to complement the theoretically derived variables, ensuring that variables showing both empirical and theoretical associations with the outcome were included in the subsequent multivariable regression model.

Group differences were tested using one-way ANOVA for continuous variables and Chi-square tests for categorical variables. Descriptive results are reported as means with standard deviations or frequencies and percentages, as appropriate.

To identify factors associated with self-reported history of falls, a multivariable logistic regression analysis was conducted. Independent variables were selected based on the correlation analyses including relevant interaction terms. Results are presented as odds ratios (ORs) with corresponding 95% confidence intervals (CIs).

Tests were two–tailed, and statistical significance was defined as a *p*-value < 0.05. Statistical analysis was performed using SPSS version 29.0.2.0 (IBM Corp).

## 3 Results

### 3.1 Demographic and socioeconomic characteristics

A total of 4,427 participants were included in the study, comprising 3,223 individuals without osteoarthritis, 880 with knee OA, 159 with hip OA, and 165 with combined hip and knee OA. Individuals with hip or combined OA were significantly older than those without OA. Women were overrepresented in the hip (71.1%) and combined OA (69.7%) groups in comparison to the knee OA (56.4%) and non-OA (58.0%) groups. Overall, 37.9% of participants were over 65 years of age, with hip OA (50.3%) and combined OA (47.9%) groups comprising the highest proportions of older individuals.

Body mass index was significantly higher in individuals with knee OA (29.5 ± 4.8) and combined OA (29.9 ± 4.9) compared to those without OA (28.2 ± 4.8; *p* < 0.001).

Participants with hip and combined OA had lower income levels than those without OA and patients with knee OA (*p* = 0.002). Table 1 presents the detailed demographic and socioeconomic characteristics of the study population.

**Table 1:** Demographic and socioeconomic characteristics of study populations.

Variables	All patients	Patients without OA	Patients with knee OA	Patients with hip OA	Patients with combined hip and knee OA	*p*-value
n	4,427	3,223	880	159	165	
Age^†^						<0.001
<65 years	2,751 (62.1%)	2,060 (63.9%)	526 (59.8%)	79 (49.7%)	86 (52.1%)	
≥65 years	1,676 (37.9%)	1,163 (36.1%)	354 (40.2%)	80 (50.3%)	79 (47.9%)	
Sex^†^						<0.001
Female	2,593 (58.6%)	1,869 (58%)	496 (56.4%)	113 (71.1%)	115 (69.7%)	
Male	1,834 (41.4%)	1,354 (42%)	384 (43.6%)	46 (28.9%)	50 (30.3%)	
BMI^*^						<0.001
	28.5 ± 4.8	28.2 ± 4.8	29.5 ± 4.8	28.2 ± 4.8	29.9 ± 4.9	
Race^†^						0.071
White people	3,502 (79.1%)	2,555 (79.4%)	684 (77.8%)	139 (87.4%)	124 (75.2%)	
African	802 (18.1%)	580 (18.0%)	167 (19.0%)	16 (10.1%)	39 (23.6%)	
American	41 (0.9%)	33 (1.0%)	7 (0.8%)	1 (0.6%)	0 (0%)	
Asian Latino	77 (1.7%)	51 (1.6%)	21 (2.4%)	3 (1.9%)	2 (1.2%)	
Marital status^†^						0.241
Living without partner	3,433 (77.6%)	2,512 (78%)	684 (77.8%)	118 (74.2%)	119 (72.1%)	
Living with partner	991 (22.4%)	709 (22%)	195 (22.2%)	41 (25.8%)	46 (27.9%)	
Income^†^						0.002
<$10K	149 (3.4%)	104 (3.5%)	28 (3.4%)	6 (4.1%)	11 (7.2%)	
$10K–$25K	423 (9.6%)	289 (9.6%)	96 (11.6%)	17 (11.5%)	21 (13.7%)	
$25K–$50K	1,053 (23.8%)	752 (25.1%)	207 (25.1%)	47 (31.8%)	47 (30.7%)	
$50K–$100K	1,493 (33.7%)	1,113 (37.1%)	273 (33.1%)	53 (35.8%)	54 (35.3%)	
>$100K	1,309 (29.5%)	743 (24.8%)	222 (26.9%)	25 (16.9%)	20 (13.1%)	
Smoking^†^						0.483
Yes	304 (6.9%)	228 (7.1%)	54 (6.2%)	8 (5.1%)	14 (8.5%)	
No	4,101 (92.6%)	2,977 (92.9%)	823 (93.8%)	150 (94.9%)	151 (91.5%)	

^*^Values are presented as mean ± standard deviation; significance tested using T-Test.

^†^Values are presented as number and percentage; significance tested using χ^2^-Test.

### 3.2 Clinical characteristics

The Charlson Comorbidity Score was significantly higher in individuals with knee OA (0.4 ± 0.9) and combined OA (0.5 ± 1.1) compared to those without OA (0.4 ± 0.8; *p* < 0.001).

Individuals with OA reported significantly poorer physical health status as measured by the SF−12 Physical Summary Scale. Scores were significantly lowest in the combined OA group (39.8 ± 10.9), followed by the knee OA group (45.8 ± 9.6), and highest in the non-OA group (50.4 ± 8.3; *p* < 0.001). The KOOS Quality of Life subscale was significantly different across OA groups (*p* < 0.001). Overall, the lowest scores were observed in participants with combined OA (46.6 ± 22.1), followed by those with knee OA (52.8 ± 19.8), while scores were higher in participants with hip OA (75.6 ± 19) and in those without OA (72 ± 21.2).

KOOS Function in Sports and Recreational Activities scores also differed significantly between groups (*p* < 0.001). The lowest scores were observed in participants with combined OA (51.3 ± 27) and knee OA (57.2 ± 26.7), while higher scores were seen in participants with hip OA (79 ± 19.9) and those without OA (77.2 ± 23.8). Depressive symptom scores (CES-D) were significantly higher in participants with osteoarthritis compared to those without OA (*p* < 0.001), with the highest values observed in the combined OA group (8.8 ± 8.9), followed by knee OA (7.7 ± 7.3) and hip OA (6.6 ± 6.3), whereas participants without OA reported the lowest scores (6.3 ± 6.8). Table 2 presents the detailed clinical characteristics of the study population.

**Table 2:** Clinical characteristics of study populations.

Variables	All patients	Patients without OA	Patients with knee OA	Patients with hip OA	Patients with combined hip and knee OA	*p*-value
n	4,427	3,223	880	159	165	
Falls^†^						0.317
Yes	1,448 (32.7%)	1,034 (32.1%)	294 (33.4%)	59 (37.1%)	61 (37.0%)	
No	2,979 (67.3%)	2,189 (67.9%)	586 (66.6%)	100 (62.9%)	104 (63.0%)	
Falls^†^						0.013
<2	3,727 (84.3%)	2,745(85.3%)	723 (82.3%)	131 (82.9%)	128 (77.6%)	
≥2	693 (15.7%)	474 (14.7%)	155 (17.7%)	27 (17.1%)	37 (22.4%)	
Charlson Comorbidity score^*^						<0.001
	0.4 ± 0.8	0.4 ± 0.8	0.4 ± 0.9	0.4 ± 0.95	0.5 ± 1.1	
SF-12: physical summary scale^*^						0.001
	50 ± 9.1	50.4 ± 8.3	45.8 ± 9.6	47.5 ± 9.3	39.8 ± 10.9	
KOOS quality of life score^*^						<0.001
	67.4 ± 22.6	72 ± 21.2	52.8 ± 19.8	75.6 ± 19	46.6 ± 22.1	
KOOS function, sports, and recreational activities^*^						<0.001
	73.1 ± 25.7	77.2 ± 23.8	57.2 ± 26.7	79 ± 19.9	51.3 ± 3	
CES-D: depression scale^*^						<0.001
	6.6 ± 7	6.3 ± 6.8	7.7 ± 7.3	6.6 ± 6.3	8.8 ± 8.9	

^*^Values are presented as mean ± standard deviation; significance tested using T-Test.

^†^Values are presented as number and percentage; significance tested using χ^2^-Test.

### 3.3 Factors associated with recurrent falls

Overall, the presence of OA diagnosis was not associated with recurrent falls; however, multivariable regression analysis identified several significant predictors of recurrent falls (Table 3). Individuals under 65 years of age exhibited reduced odds of experiencing recurrent falls compared to older individuals (*OR* = 0.752, *p* = 0.034). Marital status was also associated with recurrent fall risk, with participants living with a partner showing approximately 25% lower odds of recurrent falls compared to those living without partner (*OR* = 0.751, *p* = 0.027).

**Table 3:** Logistic regression for recurrent falls (≥2 vs. 0–1): within 12 months.

Variables	Regression coefficient	95 % confidence interval	Odds-ratio	*p*-value
Age				
<65 years				
≥65 years	−0.285	0.575–0.979	0.752	0.034
BMI				
	−0.006	0.972–1.016	0.94	0.587
Sex				
Female				
Male	−0.108	0.726–1.108	0.897	0.315
Smoking status				
Yes				
No	−0.051	0.631–1.433	0.951	0.809
Income				
<$10K				
$10K–$25K	0.278	0.685–2.545	1.321	0.406
$25K–$50K	0.410	0.817–2.776	1.506	0.189
$50K–$100K	0.416	0.819–2.806	1.516	0.185
>$100K	0.456	0.831–2.995	1.577	0.164
Marital status				
Living without partner				
Living with partner	−0.286	0.583–0.969	0.751	0.027
Race				
White people				
African American	−0.309	0.546–0.988	0.734	0.042
Asian	0.027	0.342–3.090	1.027	0.962
Latino	0.375	0.750–2.822	1.455	0.267
Charlson Comorbidity score				
	0.092	0.971–1.238	1.096	0.136
CES-D: depression scale				
	0.023	1.007–1.040	1.024	0.005
SF-12: physical summary scale				
	−0.020	0.976–0.994	0.980	0.005
KOOS quality of life score				
	−0.006	0.986–1.001	0.994	0.104
KOOS function, sports, and recreational activities				
	−0.001	0.993–1.006	0.999	0.763
Interaction age and patients with hip OA				
	−1.468	0.058–0.914	0.230	0.036
Interaction age and patients with knee OA				
	−0.087	0.052–1.613	0.917	0.763
Interaction age and patients with combined hip and knee OA				
	−0.069	0.310–2.814	0.933	0.902
Interaction depression and patients with hip OA				
	−0.045	0.880–1.039	0.956	0.292
Interaction depression and patients with knee OA				
	0.036	1.003–1.071	1.036	0.034
Interaction depression and patients combined hip and knee OA				
	0.55	0.992–1.125	1.057	0.085

All odds ratios are adjusted for covariates listed in the table (BMI, sex, smoking status, income, marital status, race, Charlson Comorbidity score, CES-D: depression scale, KOOS subscales, and osteoarthritis diagnosis) as well as the pre-specified interaction terms with age and depressive symptoms.

Regarding ethnicity, African American demonstrated significantly lower odds of recurrent falls than White people (*OR* = 0.734, *p* = 0.042), corresponding to 27% lower odds. No statistically significant differences were observed for Asian or Latino participants.

Better physical health was associated with reduced odds of recurrent falls: each one-point increase the SF−12 physical summary score was linked to a 2% decrease in odds (*OR* = 0.980, *p* = 0.005). In contrast, higher depressive symptoms measured by the CES–D scale were associated with increased odds of recurrent falls, with each additional point corresponding to a 2.4% increase in odds (*OR* = 1.024, *p* = 0.005).

A significant interaction was observed between knee OA and depressive symptoms (*OR* = 1.036, *p* = 0.034), indicating that higher depressive symptom scores were associated with higher odds of recurrent falls, particularly among participants with knee OA. Furthermore, among participants with hip OA, younger age was associated with substantially lower odds of recurrent falls (*OR* = 0.230, *p* = 0.036).

**Figure 1:**
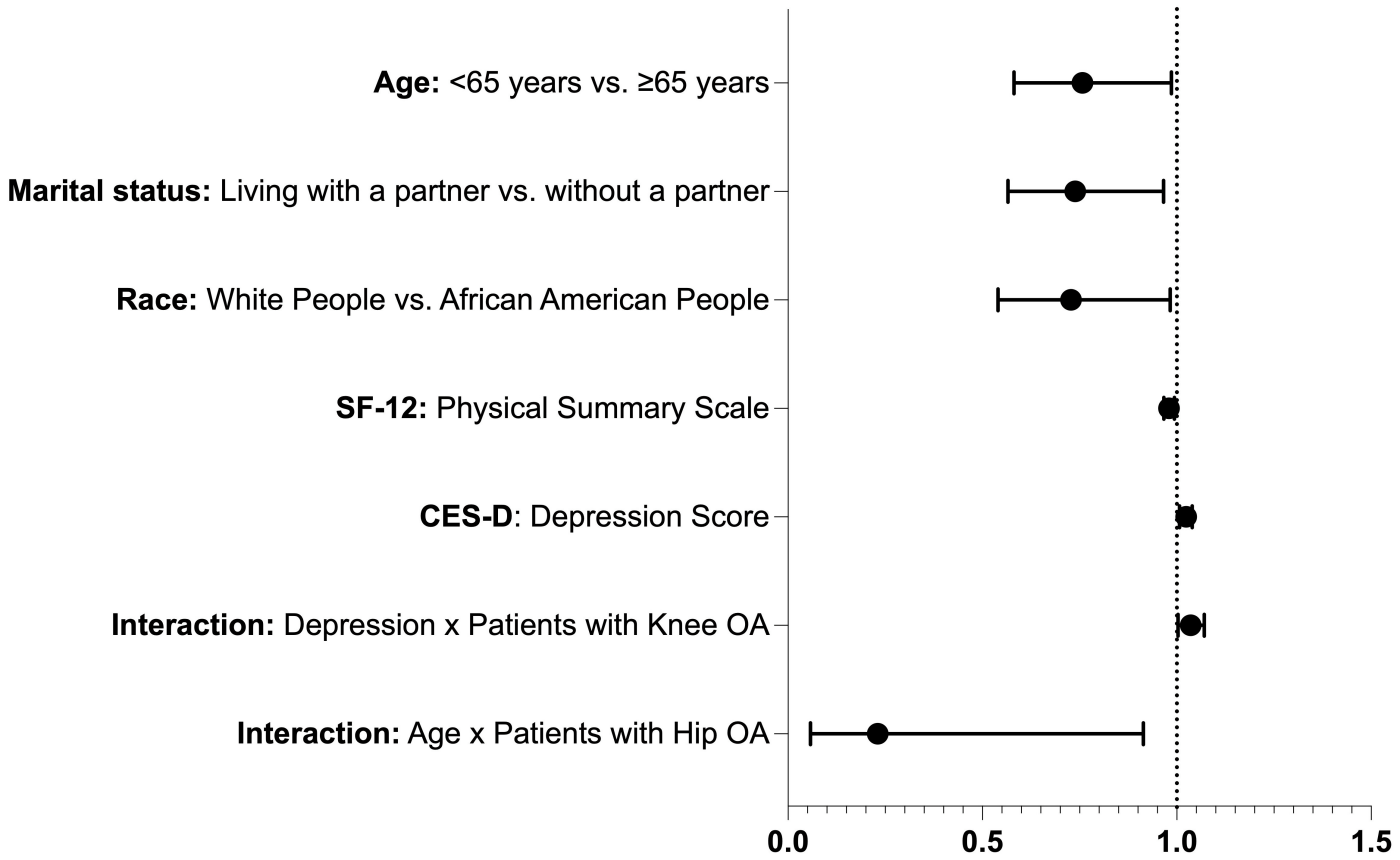
Forest plot of significant predictors of recurrent falls (≥2 falls within 12 months) from the multivariable logistic regression. Each point represents the adjusted odds ratio (OR) with 95% confidence interval (CI) on a logarithmic scale. The vertical dashed line at OR = 1 indicates no association. Predictors with OR < 1 are associated with reduced odds of recurrent falls, while predictors with OR > 1 indicate increased odds.

Figure 1 displays the forest plot of significant predictors of recurrent falls from the multivariable logistic regression. The plot visually illustrates factors associated with lower odds of recurrent falls, including younger age, living with a partner, African American ethnicity, and higher SF-12 physical scores, as well as factors associated with higher odds of recurrent falls, including higher CES-D depression scores. Interaction effects are also highlighted, showing that the association between hip OA and recurrent falls was more pronounced in older adults, while higher depressive symptom scores were associated with higher odds of recurrent falls among individuals with knee OA.

## 4 Discussion

Osteoarthritis is commonly regarded a major risk factor for falls, largely due to its association with pain, reduced joint function, and impaired mobility (5, 22, 25). Our findings challenge the assumption that an osteoarthritis diagnosis alone increases the risk of recurrent falls. After adjusting for demographic, functional, and psychological factors, OA was not independently associated with recurrent fall risk. Only hip OA demonstrated a non-significant trend toward increased recurrent fall risk, whereas knee OA and combined hip and knee OA were not associated with recurrent falls. Instead, recurrent falls risk were associated with a complex interplay of age, marital status, ethnicity, physical functioning, and depressive symptoms, with specific interactions involving OA location. Specifically, depressive symptoms were associated with higher odds of recurrent falls in individuals with knee OA. Moreover, among those with hip OA, younger adults exhibited substantially lower odds of recurrent falls compared to older adults, suggesting that the association between hip OA and recurrent falls was more pronounced in older individuals. The 12-month follow-up period was deliberately selected to minimize recall bias and reduce sample attrition, common challenges in longer-term studies involving older adults. This design allowed us to more precisely capture the short-term influence of psychological health, functional changes, and comorbidities on fall risk, supporting a shift from a disease-centered to a multifactorial perspective in OA-related fall prevention.

Age remains one of the strongest non-modifiable risk factors for falls. In our study, participants under 65 years had significantly lower odds of recurrent falls than those aged ≥65 (*OR* = 0.75, p = 0.034), indicating about 25% lower risk, consistent with well-established epidemiological evidence. Increasing age has been associated with impaired proprioception, reduced neuromuscular response, and slower gait speed, all of which contribute to postural instability in older adults (6, 22, 26). Findings based on another study demonstrated that newly diagnosed OA patients over age 65 had a disproportionately higher fall incidence than younger individuals, despite similar radiographic severity (26).

In this study we observed a significant interaction between age and hip OA: among participants with hip OA, being younger markedly reduced the risk of recurrent falls, suggesting that the increased fall risk related to hip OA predominantly affects older adults. This is consistent with biomechanical data showing that hip OA contributes to asymmetric loading and reduced abduction strength in older adults (23, 27).

Sex was not independently predictive of recurrent falls. Women in our cohort were more frequently disproportionately affected by hip and combined OA and showed lower physical and mental health scores, but these factors did not translate into an independent effect of sex on recurrent falls. The observed gender differences in functional and psychological burden may still contribute indirectly to fall vulnerability (28, 29).

Marital status was a significant predictor of recurrent falls: living with a partner was associated with 25% lower odds of recurrent falls compared to those living without partner (*OR* = 0.75, *p* = 0.027), consistent with a protective role of social support. A South African cohort of long-term care residents found that married elders fell less often than single or widowed peers (30). In another hip and/or knee OA cohort study, living alone was a significant predictor of falls (31), and the English Longitudinal Study of Aging reported higher fall rates in women who had never married (32).

Furthermore, ethnicity emerged as a relevant factor, with African American showing 27% lower odds of recurrent falls compared to White people. In a nationally representative US cohort (NHATS), Black seniors had 30%−40% lower risk of any and recurrent falls compared to White people, even after adjusting for comorbidities (33). Cultural, behavioral, and environmental factors may help explain these disparities and should be considered in the development of fall prevention strategies. These findings highlight the importance of culturally tailored interventions that address the specific social and behavioral contexts of diverse populations.

Lower physical functioning was significantly associated with recurrent falls in individuals with osteoarthritis. Lower scores on the SF-12 physical summary were independently associated with increased odds of recurrent falls, highlighting the importance of preserving physical capacity to prevent falls. This aligns with prior research identifying impaired muscle strength, balance, and gait speed as some of the most powerful modifiable predictors of falls in OA populations (34–36). For example, van Schoor et al. using data from EPOSA cohort, reported that reduced physical function explained a substantial portion of the fall risk associated with clinical OA (12). Consequently, rehabilitation strategies that emphasize progressive resistance training, balance improvement, and functional mobility should be prioritized in OA management to reduce fall risk.

In addition, psychological factors, particularly depressive symptoms, also significantly contributed to fall susceptibility. Higher CES-D Scale scores were associated with increased odds of recurrent falls, and depressive symptoms specifically amplified fall risk in participants with knee OA. This echoes prior evidence from previous studies, showing that psychological distress and fear of falling are strong independent predictors of falls among individuals with in OA (18, 23). Depression can impair executive function, reduce physical activity, and increase frailty, all of which mediate fall risk. As such, routine screening and targeted treatment of depressive symptoms should be incorporated into comprehensive OA care pathway.

Several limitations in this study must be acknowledged when interpreting our findings. First, falls were self-reported and may be affected by recall bias, particularly in older adults. Although the 12-month follow-up period was deliberately selected to balance clinical relevance with recall accuracy, misreporting of fall events cannot be fully excluded. In addition, OA subgroup classification was based on physician diagnosis and radiographic evidence; however, some degree of misclassification cannot be excluded, particularly given symptoms overlap between hip and knee OA, Thirdly, some fall–related contributors, such as cognitive status, home environment, medication use, and fear of falling, were not captured in this dataset. Lastly, because our sample was composed of ambulatory, community-dwelling adults in the United States, generalizability to other populations may be limited.

In conclusion, our findings challenge the prevailing notion that osteoarthritis itself is the primary cause of recurrent falls. This study is the first to demonstrate, that in individuals with newly diagnosed hip and/or knee osteoarthritis, recurrent fall risk over a 12-month period is not explained by the structural diagnosis alone. Instead, its risk is shaped by a multifactorial interplay of age, marital status, ethnicity, physical functioning, and depressive symptoms, with specific interactions involving OA location, particularly between age and hip OA, and between depression and knee OA. For clinical practice, this means fall prevention in OA should move beyond joint-focused care. Screening protocols should routinely include assessment of depressive symptoms, and functional performance like balance or gait deficits. Targeted interventions, such as structured strength and balance programs, and integration of mental health support, should become part of standard OA management. By addressing these modifiable contributors of aging, clinicians can meaningfully reduce recurrent falls and improve safety and independence in this growing patient population.

These findings refine the understanding of fall risk in OA and highlight the need for future research and clinical approaches that address the interplay of physical, psychological, and demographic factors, rather than attributing fall risk solely to joint pathology.

## Data Availability

Publicly available datasets were analyzed in this study. This data can be found here: Osteoarthritis Initiative https://nda.nih.gov/oai.
