# Supplementary material for: Multivariate analysis of newly diagnosed hip, knee, and combined hip and knee Osteoarthritis and recurrent fall risk: data from the Osteoarthritis Initiative
Source: Front Med (Lausanne). 2026 Apr 10;13:1710951. doi: 10.3389/fmed.2026.1710951 (PMC13106385; doi:10.3389/fmed.2026.1710951)
Supplement: Supplementary file 1 [file Table_1.docx]

Supplementary Table. Correlation analyses (r, P value)

|  |  | Patients without OA | Patients with Knee OA | Patients with Hip OA | Patients with Knee and Hip OA |
| --- | --- | --- | --- | --- | --- |
| Age | r  p value | -0.081  <0.001 | 0.031  0.042 | 0.072  <0.001 | 0.055  <0.001 |
| Age <65 years old vs. >65 years old | χ²  p value | 15.856  <0.001 | 2.619  0.106 | 10.876  <0.001 | 7.315  0.007 |
| BMI | r  p value | -0.104  <0.001 | 0.104  <0.001 | -0.001  0.970 | 0.076  <0.001 |
| Gender | χ²  p value | 1.660  0.198 | 2.208  0.137 | 10.614  <0.001 | 8.741  0.003 |
| Income | r  p value | 0.037  0.018 | 0.004  0.817 | -0.32  0.040 | -0.063  <0.001 |
| Charlson Comorbidity Score | r  p value | -0.052  <0.001 | 0.051  0.001 | 0.011  0.517 | 0.033  0.059 |
| SF-12: Physical Summary Scale | r  p value | 0.246  <0.001 | -0.210  <0.001 | -0.072  <0.001 | -0.209  <0.001 |
| KOOS Quality of Life Score | r  p value | 0.338  <0.001 | -0.358  <0.001 | 0.033  0.054 | -0.223  <0.001 |
| KOOS Function, Sports, and Recreational Activities | r  p value | 0.282  <0.001 | -0.289  <0.001 | -0.002  0.932 | 0.180  <0.001 |
| CES-D: Depression Scale | r  p value | -0.060  <0.001 | 0.046  0.003 | 0.022  0.194 | 0.058  <0.001 |
| Recurrent falls:  0-1 fall vs. 2-4 falls | χ²  p value | 6.212  0.013 | 3.654  0.056 | 0.057  0.811 | 4.028  0.045 |
